# Epigenome-wide discovery and evaluation of leukocyte DNA methylation markers for the detection of colorectal cancer in a screening setting

**DOI:** 10.1186/s13148-017-0322-x

**Published:** 2017-03-03

**Authors:** Jonathan Alexander Heiss, Hermann Brenner

**Affiliations:** 1 Division of Clinical Epidemiology and Aging Research, German Cancer Research Center (DKFZ), Im Neuenheimer Feld 581, Heidelberg, 69120 Germany; 2 Division of Preventive Oncology, National Center for Tumor Diseases (NCT) and German Cancer Research Center (DKFZ), Heidelberg, Germany; 3 German Cancer Consortium (DKTK), German Cancer Research Center (DKFZ), Heidelberg, Germany

**Keywords:** Biomarker, Colorectal cancer, Early detection, Screening setting, DNA methylation, Illumina Infinium 450K, Epigenome-wide association study, EWAS, Leukocyte composition

## Abstract

**Background:**

Colorectal cancer (CRC) is the third most common cancer worldwide. If detected at an early stage, prognosis is good. Despite increasing evidence for the benefits of implemented screening programs, such as screening colonoscopy, compliance is rather low. Hence there is demand for non-invasive tests for the early detection of CRC with high acceptance in population-wide screening. The objective of this study was to identify and evaluate leukocyte DNA methylation patterns as a potential biomarker for early detection of CRC.

**Methods:**

Blood samples of patients scheduled for a screening colonoscopy were collected before the procedure. Additionally, blood samples from CRC cases recruited in a clinical setting were collected. DNA was extracted from leukocytes, and DNA methylation was measured with the Infinium 450K BeadChip. In total, 46 CRC cases and 140 controls from the screening setting and 93 CRC cases from the clinical setting were measured.

**Results:**

An epigenome-wide discovery revealed two CpG sites in the promoter region of KIAA1549L that were significantly differentially methylated between cases and controls. A third marker in the body region of BCL2 was discovered in a candidate approach testing biomarkers reported in the literature. Logistic regression models built on these three markers yielded an optimism-corrected *c*-statistic of 0.69 in the screening setting and 0.73 in the clinical setting.

**Conclusions:**

Although diagnostic performance of the DNA methylation signature identified in this first epigenome-wide association study of leukocyte DNA methylation with CRC in a screening setting is not competitive with established screening tests, the identified markers may contribute to multimarker panels for early detection of CRC.

**Electronic supplementary material:**

The online version of this article (doi:10.1186/s13148-017-0322-x) contains supplementary material, which is available to authorized users.

## Background

With ∼1.4 million incident cases and almost 700,000 deaths in 2012, colorectal cancer (CRC) is the third most common cancer and the fourth most common cause of cancer death worldwide [1]. Stage at diagnosis is the most important prognostic factor with relative 5-year survival rates of 90, 74, and 14% when diagnosed at a localized, regional, and advanced stage, respectively [2]. Colonoscopy is the gold standard for the detection of CRC and its precursors. CRC is diagnosed earlier by screening colonoscopy [3] and can even be prevented by removing precursors during colonoscopy. In a recent meta-analysis, screening colonoscopy was estimated to reduce the risk of incident CRC by 69% and CRC mortality by 68% [4].

In Germany, a first screening colonoscopy is offered free of charge to men and women aged 55 and older, a second one is possible after 10 years. Alternatively, biennial fecal occult blood tests can be performed. Nearly 90% of the people entitled were found to be aware of this program [5], but despite awareness and clear benefits, less than 40% were up to date with CRC screening recommendations [6]. A recent study among 172 asymptomatic participants recruited during regular consultations found that 109 (63%) refused to undergo screening colonoscopy, but of these, 106 (97%) accepted non-invasive screening methods with 90 (83%) choosing a blood-based test [7]. Although blood tests do not achieve the excellent performance of colonoscopy or immunochemical fecal occult blood tests, their value comes from the high acceptance by patients. Higher participation in screening programs could lower CRC mortality.

Only a fraction of the biomarkers for the early detection of cancer reported in the literature find their way to clinical application eventually. Promising results from retrospective case-control studies often cannot be validated in prospective studies. A great number of blood-based biomarkers for the early detection of CRC have been reported [8], but only one has been approved by the U.S. Food and Drug Administration so far: the Epi proColon test (Epigenomics AG, Berlin, Germany), that measures the methylation of the SEPT9 gene in circulating cell-free DNA in blood, detected CRC with a sensitivity (specificity) of 48% (92%) in a screening setting [9]. Several studies have reported leukocyte DNA methylation (DNAm) markers for various types of cancer (Additional file 1: Table S1), but none of these studies were conducted in a screening setting. We used blood samples from BLITZ (German: Begleitende Evaluierung innovativer Testverfahren zur Darmkrebs-Früherkennung), a study among participants of screening colonoscopy, to conduct the first epigenome-wide association study (EWAS) for leukocyte DNAm markers for the early detection of CRC in a screening setting.

## Methods

### Study population

We analyzed blood samples collected in a screening setting (BLITZ study) and in a clinical setting (DACHS^+^ study). Details of both studies have been described elsewhere [10, 11]. In brief: BLITZ is conducted in cooperation with several gastroenterological practices in Southern Germany. Eligible are men and women aged 55 to 75 who are scheduled for a screening colonoscopy. They are informed about the study and invited to participate by their physicians at a preparatory visit for the colonoscopy. Participants are excluded, if colonoscopy is indicated due to other reasons (e.g., visible rectal bleeding or a positive test for fecal occult blood), if they had a previous endoscopic examination within the preceding 5 years, or if they had a previous gastrointestinal cancer diagnosis. Blood samples are taken before the colonoscopy; afterwards, medical reports are obtained from the physicians. Recruitment is ongoing; 6613 participants had been enrolled by the end of 2014. They were classified according to the most advanced findings as follows: CRC (*n* = 57), advanced adenomas, non-advanced adenomas, undefined polyps or other findings of the colonic mucosa (such as pseudopolyps), hyperplastic polyps (*n* = 643), neither of these findings (*n* = 3856). For this analysis, we selected CRC cases and participants of the last two categories as controls; all other categories were excluded.

Because of the limited number of CRC cases even in such a large screening study, CRC cases recruited in a clinical setting in the DACHS^+^ study were included in addition. In the DACHS^+^ study, 819 men and women aged 55 to 75 with a first diagnosis of a gastrointestinal cancer between October 2006 and December 2014 from several clinics in SouthWest Germany were recruited. Patients with a previous cancer diagnosis in the gastrointestinal tract were excluded. Blood samples were collected before surgery.

### Sample selection and processing

**Fig. 1:**
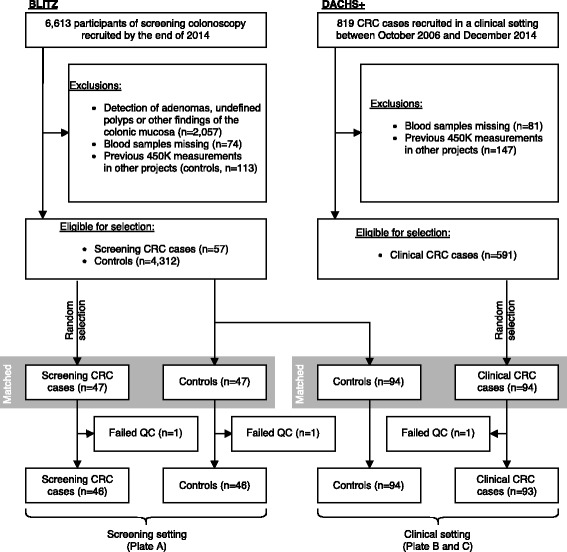
Sample selection. Blood samples from CRC cases and controls free of colorectal neoplasms collected in a screening setting (BLITZ) and from CRC cases collected in a clinical setting (DACHS^+^) were used. A selection of samples matched for sex and age were measured on the Illumina Infinium 450K chip. The final numbers of samples used for the current analysis, after the exclusion of three samples with unreliable measurements (QC, quality control), are indicated at the *bottom*

For the current analysis, blood samples from 47 CRC cases and 141 controls from BLITZ and 94 CRC cases from DACHS^+^ were used. An overview of the selection procedure is depicted in Fig. 1. DNA was extracted from buffy coat samples and DNA methylation was measured on the Infinium HumanMethylation450 BeadChip (Illumina, San Diego, CA, USA) (450K) at the Genomics and Proteomics Core Facility at the German Cancer Research Center, Heidelberg, Germany, according to the manufacturer’s instructions. This platform queries the methylation levels of 485,512 CpG sites. To minimize potential impact of batch effects, samples were allocated to three 96-well plates as follows: plate A with randomly selected screening CRC cases and controls from BLITZ 1:1-matched for sex and age; plate B with randomly selected clinical CRC cases from DACHS^+^ and controls from BLITZ 1:1-matched for sex and age; and plate C as plate B. We will refer to the samples on plate A as screening setting and to the samples on plates B and C as clinical setting.

### Epigenome-wide marker discovery

450K data were normalized using the R package normalize450K [12]. Methylation levels of each CpG site were regressed linearly on disease status and the following covariates: sex, age, leukocyte composition, and batch (96-well plate on which samples were run). Methylation levels were expressed as *β*-values because of their linear relation with cell proportions. Cell proportions of six major leukocyte types were estimated according to Houseman et al. [13], including granulocytes, monocytes, CD8+ T-cells, CD4+ T-cells, natural killer cells, and B lymphocytes. Disease status was coded by two variables, *screening* and *clinical*, in such a way that screening cases and clinical cases were compared only to controls within the same setting. We compared this first model to a second one without *screening* and *clinical* and used the likelihood-ratio test to assess their significance. CpG sites that were significantly associated at a false discovery rate of 10% and showed a consistent trend in the screening and clinical setting (same sign for regression coefficients *r*_*s*_ and *r*_*c*_ of *screening* and *clinical*) were selected as markers.

### Literature search

In addition to the hypothesis-free approach, we also deployed a targeted approach. We searched PubMed for publications from January 2011 to June 2016 reporting leukocyte DNAm markers for any kind of solid cancer. We found 18 relevant publications which reported either probe identifier (in case they used the 27K or 450K platform) or genes, in which case they were mapped to probes on the 450K chip using the annotation provided by the manufacturer. Five publications reported 14 probe identifiers and 13 publications reported 32 genes mapping to 733 probes. Association of these in total 747 probes with CRC was tested using the same approach as in the epigenome-wide discovery. Search terms and a list of all included publications are provided in Additional file 1: Table S1.

### Fitting diagnostic models

*β*-values of the markers discovered in the previous steps were adjusted for leukocyte composition and batch effects by subtracting related terms from the corresponding linear regression models to compute *β* ^′^. Three different models for CRC diagnosis were trained by logistic regression: (i) a risk-factors-only model that included only the risk factors sex and age; (ii) a markers-only model that included only *β* ^′^-values of the epigenetic markers; and (iii) a full model, including both markers and risk factors. To avoid overoptimism and to provide 95% confidence intervals, we generated 1000 stratified bootstrap samples (“stratified” meaning, that the number of cases and controls was the same as in the original sample). Models were fitted on the bootstrap samples and tested on the left-out subjects. This was done separately for the screening setting (plate A) and the clinical setting (plates B and C). Discrimination was measured by the *c*-statistic.

### Estimating model performance in the BLITZ population

The improvement in discrimination in the matched case-control sample by the full model compared to the risk-factors-only model does not represent the improvement in discrimination in the BLITZ population. Matching eliminated the association between CRC and the risk factors sex and age. We used the approach described in [14] to correct and weight the scores from our logistic regression models. Let *Y*_*i*_ be the outcome (case/control), *X*_*i*_ the biomarker levels, and *Z*_*i*_ the other risk factors for subject *i*. To correct the log odds ln*O*(*Y*_*i*_|*X*_*i*_,*Z*_*i*_)^*M*^ from the logistic regression model trained on the matched case-control sample *M*, subtract ln*O*(*Y*_*i*_|*Z*_*i*_)^*M*^, the log odds from the risk-factors-only model, and add ln*O*(*Y*_*i*_|*Z*_*i*_), the log odds from the risk-factors-only model trained on the entire BLITZ population.

$$\begin{array}{*{20}l} \ln O(Y_{i}|X_{i},Z_{i})'=&\ln O(Y_{i}|X_{i},Z_{i})^{M}-\ln O(Y_{i}|Z_{i})^{M} \\ &+\ln O(Y_{i}|Z_{i})  \end{array} $$

The result should now reflect the impact of the risk factors. To account for the different distribution of *Z* in the population than in the matched sample, subjects were weighted by inverse propensity scores (a logistic regression with the dependent variable indicating if a subject is included in the matched sample and *Z* as predictors), so that the weighted matched sample reflected the distribution of *Z* in the BLITZ population. Discrimination was measured by the weighted *c*-statistic. Performance of the other models in the BLITZ population was estimated analogously.

## Results

Three 450K assays failed the quality control (one case from each setting and one control). Study population characteristics of the remaining participants are presented in Table 1. The same table also lists the estimated average leukocyte proportions among screening cases, clinical cases, and controls. Differences in cell proportions were larger between clinical cases and controls than between screening cases and controls for all cell types. This shows that leukocyte composition is indeed an important potential confounder, even more so in the clinical setting.

**Table 1:** Study population characteristics

	Cases	Controls	
BLITZ population			
Sex (m/f)	38/19	1,978/2,521	
Mean age ± SD	67 ± 7	62 ± 7	
Smoking (never/ever)	25/32	2,313/2,175/11^a^	
Stage (Tis/I/II/III/IV)	4/19/6/19/3/6^a^	—	
Screening setting (plate A)			
Study	BLITZ	BLITZ	
Sex (m/f)	30/16	29/17	
Mean age ± SD	67 ± 7	67 ± 7	
Smoking (never/ever)	21/25	25/21	
Stage (Tis/I/II/III/IV)	2/15/5/16/3/6^a^	—	
Cell proportions			|*Δ*|
Granulocytes	54.9 ± 12.8	48.7 ± 14.9	6.2
Monocytes	7.2 ± 1.9	7.6 ± 2.8	0.4
Natural killer cells	11.7 ± 5.2	13.3 ± 5.8	1.7
CD8+ T cells	3.5 ± 4.1	4.4 ± 3.8	0.9
CD4+ T cells	16.7 ± 7.7	19.1 ± 9.5	2.3
B lymphocytes	5.9 ± 2.8	6.8 ± 3.9	0.9
(Difference in mean LC is not significant, *p*-value 0.29)^c^			
Clinical setting (plates B and C)			
Study	DACHS^+^	BLITZ	
Sex (m/f)	57/36	57/37	
Mean age ± SD	65 ± 8	65 ± 8	
Smoking (never/ever)	43/50	47/47	
Stage (Tis/I/II/III/IV)	4/28/41/29/22/1^a^	—	
Neoadj. therapy (none/rad./chemo./comb.)^b^	60/12/2/19	—	
Cell proportions			|*Δ*|
Granulocytes	64.9 ± 12.8	52.3 ± 12.6	12.6
Monocytes	9.3 ± 3.8	7.8 ± 2.7	1.5
Natural killer cells	7.2 ± 4.3	11.1 ± 5.2	3.9
CD8+ T cells	2.0 ± 2.9	3.5 ± 3.9	1.5
CD4+ T cells	12.6 ± 7.1	19.4 ± 7.8	6.9
B lymphocytes	4.0 ± 2.6	5.9 ± 2.9	1.9
(Difference in mean LC is significant, *p*-value 3.9 × 10^−11^)^c^			

Study population characteristics stratified by plate. Subjects who smoked regularly more than 1 year of their life were defined as *ever* smokers. |*Δ*| gives the absolute difference in average leukocyte proportions between cases and controls

^a^Missing values

^b^Neoadjuvant therapy (none/radiation/chemotherapy/combination of radiation and chemotherapy)

^c^As cell proportions are compositional data, differences between cases and controls should not be tested for individual cell types, but for the composition as a whole. The isometric log-ratio transformation was applied and differences were tested using multivariate analysis of variance (MANOVA) as described in [25]

At a false discovery rate of 10%, there was a single significant hit (without adjustment for LC, there would have been 90,288), cg04036920, located 1373 basepairs upstream to the transcription start site of KIAA1549L. Methylation levels of proximal CpG sites are often correlated; therefore, we tested the remaining 25 probes on the 450K chip that are ascribed to KIAA1549L, in case we missed some of them due to the high multiple-testing burden. After Bonferroni correction (significance level *α*<0.05/25), one other site was significant, cg14472551, which is located 557 basepairs apart from cg04036920 and 817 basepairs upstream to the transcription start site. Cases showed higher methylation levels than controls consistent between screening and clinical setting for both markers (cg04036920 *r*_*s*_=0.029,*r*_*c*_=0.032; cg14472551 *r*_*s*_=0.042,*r*_*c*_=0.022). Two more probes were significant at a false discovery rate of 10% when we tested only the candidates extracted from our literature search (without adjustment for LC there would have been 170). One was excluded, as *r*_*s*_ and *r*_*c*_ showed opposite trends. The other probe, cg12459502 (*r*_*s*_=0.011,*r*_*c*_=0.025), located in the body region of BCL2, was added to the marker panel. Based on nine technical replicates of a single sample, allocated on the same plates, we estimated standard measurement errors of 0.029, 0.019, and 0.019 for cg04036920, cg14472551, and cg12459502, respectively.

Thirty-three cases from DACHS^+^ received neoadjuvant therapy before blood sampling. In a sensitivity analysis, we excluded these cases (but kept the matched controls). While cg04036920 was no longer significant after correction for multiple testing in the epigenome-wide search, it remained the marker with the smallest *p*-value. The targeted search yielded again cg12459502, this time as the only significant marker.

**Fig. 2:**
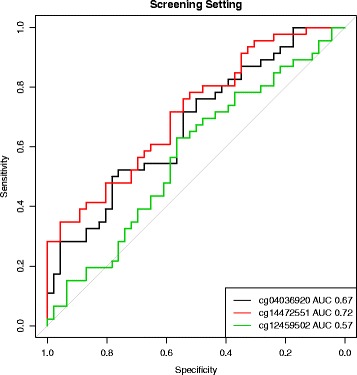
Single marker performance in screening setting. ROC curves of the three markers after correction of *β*-values for leukocyte composition and batch effects

**Fig. 3:**
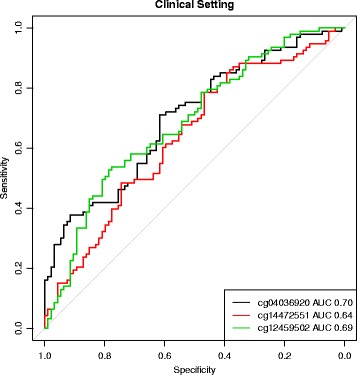
Single marker performance in clinical setting. ROC *curves* of the three markers after correction of *β*-values for leukocyte composition and batch effects

All three markers were associated with leukocyte composition, meaning that some of the regression coefficients assigned to cell proportions were highly significant. However, due to the nature of compositional data, variability in whole blood DNAm levels cannot be assigned to individual cell types. Additional file 1: Figure S1 shows the methylation levels of these markers in seven purified leukocyte types. Table 2 shows the single marker performance after adjusting *β*-values for leukocyte composition and batch effects. ROC curves are provided in Figs. 2 and 3.

**Table 2:** Single marker performance in matched case-control sample

Marker	Screening setting	Clinical setting
cg04036920	0.67	0.70
cg14472551	0.72	0.64
cg12459502	0.57	0.69

Discrimination of CRC cases and controls by single markers measured by the *c*-statistic in the screening and clinical setting. Methylation levels were adjusted for leukocyte composition and batch effects

Diagnostic models based on these three markers and the risk factors sex and age were trained and evaluated via bootstrapping. Table 3 shows the results separately for the screening setting and the clinical setting. *c*-statistics of the risk-factors-only models were around 0.5, as matching eliminated the association of sex and age with CRC. The risk-factors-only models reached *c*-statistics of 0.68 and 0.74 after weighting the matched samples for the screening setting and clinical setting, respectively. The full models did not perform better than the markers-only models in the matched samples, as expected, but also in the population setting did the combination of markers and risk factors barely improve discrimination compared to the markers-only models. Respective *c*-statistics in the clinical setting were all higher, going up to 0.81 for the full model.

**Table 3:** Performance of diagnostic models

Model	Matched sample	BLITZ population
Screening setting		
Risk-factors-only model	0.43 (0.29,0.55)	0.68 (0.57,0.76)
Markers-only model	0.69 (0.55,0.82)	0.74 (0.57,0.87)
Full model	0.69 (0.54,0.83)	0.76 (0.61,0.87)
Clinical setting		
Risk-factors-only model	0.45 (0.35,0.54)	0.74 (0.70,0.77)
Markers-only model	0.73 (0.63,0.83)	0.78 (0.66,0.88)
Full model	0.73 (0.61,0.82)	0.81 (0.71,0.88)

Mean and 95% confidence intervals of *c*-statistics. Values for “BLITZ population” represent a weighting of participants from the matched sample to achieve similar distribution of risk factors as in the BLITZ population. The three models are: (i) risk-factors-only model including sex and age; (ii) markers-only model, including methylation levels of the three identified biomarkers; and (iii) full model, combining both risk factors and markers

## Discussion

We analyzed blood samples of CRC cases and controls collected prospectively from participants of screening colonoscopy. Additionally, blood samples from clinical CRC cases were collected. An epigenome-wide discovery of leukocyte DNAm markers for the early detection of CRC identified two differentially methylated CpG sites located near the transcription start site of KIAA1549L. A third marker in the body region of BCL2 was identified in a targeted approach looking only at candidate markers reported in the literature. Discrimination (measured by the *c*-statistic) of CRC cases and controls by logistic regression models based on these three markers was 0.69 and 0.73 in the screening setting and clinical setting, respectively, and was estimated at 0.74 for the target population of the German CRC screening program. To our knowledge, this is the first epigenome-wide association study (EWAS) for leukocyte DNAm markers for the early detection of CRC in a screening setting.

Screening colonoscopy, the gold standard for the detection of CRC and precursors, suffers from low adherence. Non-invasive tests could increase participation in CRC screening programs and interest in the identification of suitable biomarkers is growing [8], but only a few were validated in a screening setting: the most advanced blood test so far, based on the detection of cell-free methylated SEPT9 in plasma, achieved a sensitivity (specificity) of 48% (92%) for the detection of CRC [9]. The combination of two other markers, CEA and anti-TP53 antibody, also evaluated in the BLITZ study (albeit not using the same samples as in the current analysis), achieved a sensitivity (specificity) of 58% (90%) [15].

Stool tests represent another non-invasive alternative. The Cologuard test (Exact Sciences, Madison, WI, USA) combines assays to test for aberrant methylation of the promoter regions of the BMP3 and NDRG4 genes, for mutations of the KRAS gene, and for human hemoglobin. A score calculated from the combined results from these assays led to an improved sensitivity (but decreased specificity) of 92% (87%) compared to 72% (95%) when using only the hemoglobin component [16]. In the BLITZ study, a standalone fecal immunochemical occult blood test (FIT) had a sensitivity (specificity) of 73% (96%) [17]. FITs perform so far substantially better than blood-based tests.

Our study has several strengths and limitations. Most importantly, we are using CRC cases and controls from a true screening setting. Blood samples in BLITZ were collected before the participants were aware of their case/control status, thereby eliminating (largely) the possibility of selection bias and information bias. Case-control studies using clinical settings often try to account for selection bias by matching cases and controls on a number of confounders. However, there might be unknown confounders which cannot be accounted for and which might result in false positive candidate markers. Using a screening setting ensures that cases and controls are (on expectation) comparable even for the unknown confounders. For instance, Prolactin, discovered in a clinical setting as a biomarker for ovarian cancer, failed in a screening setting later and turned out to be sensitive to the way samples were collected (at the day of a planned surgery or in the days before) and might just be a symptom of stress [18, 19]. There might be many ways in which patients change their lifestyle after a cancer diagnosis. In such cases, not the presence of the disease but being aware of it might cause differences in biomarker levels. Using a screening setting avoids these pitfalls. Of course, these considerations do not apply to the DACHS^+^ study. Therefore, we filtered out candidates that showed a inconsistent trend in the screening and clinical setting. Furthermore, the BLITZ population closely resembles the target population, as should the AUC estimates of the diagnostic models.

Another strength of our study is the high sample size. BLITZ is one of the largest screening studies for CRC with more than 6600 participants. Therefore, despite the low prevalence of CRC in the BLITZ population (<1%), we had approximately 60 samples of CRC to choose from. We still might have missed potential markers due to a lack of statistical power caused by the high multiple testing burden. We compensated for this by including samples from clinical CRC cases, but, as outlined above, they are no equivalent substitute.

We matched cases and controls on the risk factors sex and age. Matching would not be necessary in BLITZ, as the study population closely mirrors the target population, in which these groups differ on these factors, and might even lead to biased estimates of sensitivity and specificity if marker levels are associated with the factors matched on [20, 21]. Matching was done here to increase the statistical power to find biomarkers that provide diagnostic value beyond these known risk factors. We used the method described in [14] to arrive at presumably unbiased estimates of marker performance in the BLITZ population.

Another strength of our study is the adjustment for leukocyte composition (LC). LC is often considered as the most important confounder when analyzing whole blood DNAm [22]. This turned out to be true here as well. Clinical cases and controls differed much more than screening cases and controls, which may reflect the higher fraction of late stages among clinical cases and the fact that some received neoadjuvant therapy. Confounding by LC might be one of the reasons, why none save one candidate marker from our literature search could be validated here. Only two of the studies included did adjust for LC, and none was conducted in a screening setting (with the exception of one study looking at colorectal adenomas). Indeed, an unadjusted analysis of our data would have confirmed 170 of the 747 markers. Another reason might be that those markers are specific for the types of cancer investigated in the original studies.

We adjusted for six major leukocyte types, yet we cannot exclude the possibility that observed differences at the identified markers are still due to residual confounding, either due inaccurate cell proportion estimates or because an even finer distinction of cell types would be necessary. On the other hand, regardless if the observed effects represent genuine changes of the methylation state or not, a biomarker must merely hold predictive value. One could use not only the marker panel but also harness the predictive value of the leukocyte composition, as proposed in [23]. Again, as seen in the differences in leukocyte composition between screening and clinical cases, such markers would need to be evaluated in a screening setting. A detailed investigation of this issue was beyond the scope of this work. However, metrics like the neutrophil/lymphocyte ratio are influenced by many factors and therefore cannot be specific for CRC.

It is unlikely that observed differences between cases and controls are caused by batch effects. Blood samples were collected blinded to the outcome, as well were DNA extraction and DNAm measurements. Batch effects are omnipresent and numerous for the 450K platform [24], but our careful sample allocation scheme ensured that they were not associated with the outcome or the matching factors.

It is unclear if the identified methylation signature is specific for CRC, as in contrast to cell-free DNA in blood serum which might originate from tumor tissue, changes in leukocyte DNA methylation probably reflect a response of the immune system which might be similar for other cancers or diseases.

The function of KIAA1945L is largely uncharacterized; therefore, we refrain from speculations about the biological plausibility of this finding.

## Conclusions

We identified three CpG sites whose methylation levels in whole blood can be used as biomarkers for the early detection of colorectal cancer. While their performance on their own is not competitive to screening colonoscopy or fecal immunochemical tests, their combination in a multi-marker panel, similar to the multitarget stool test mentioned above [16], could render them useful as a screening tool. Further validation of these markers in other study populations is necessary.

## Additional file

Additional file 1 Supplement.pdf. Includes Figure S1, PubMed search terms, Table S1 and supplementary references. (PDF 53 kb)
