# Supplementary material for: Epigenome-wide discovery and evaluation of leukocyte DNA methylation markers for the detection of colorectal cancer in a screening setting
Source: Clin Epigenetics. 2017 Mar 3;9:24. doi: 10.1186/s13148-017-0322-x (PMC5335821; doi:10.1186/s13148-017-0322-x)
Supplement: Additional file 1 — Supplement.pdf. Includes Figure S1, PubMed search terms, Table S1 and supplementary references. (PDF 53 kb) [file 13148_2017_322_MOESM1_ESM.pdf]

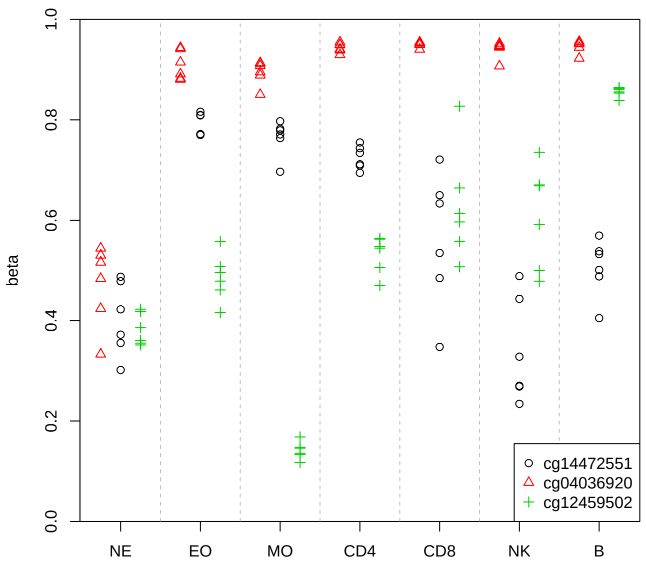

Figure S1 Marker methylation levels in purified leukocytes. Plotted are β-values of the identified markers in seven cell types isolated from blood samples of six healthy men Reinius et al. (2012). Abbreviated cell types: NE neutrophils, EO eosinophils, MO monocytes, CD4+ T cells, CD8+ T-cells, NK natural killer cells, B CD19+ B lymphocytes.

PubMed search terms:

```
(
        "Early Detection of Cancer"[MeSH Heading]
        AND "DNA Methylation"[MeSH Heading]
        AND "Biological Markers"[MeSH Heading]
)
OR (
                (
                "cancer"[Title/Abstract]
                OR "neoplasms"[Title/Abstract]
                OR "malignancies"[Title/Abstract]
                OR "carcinoma"[Title/Abstract]
                )
        AND (
                "blood"[Title/Abstract]
                OR "leukocytes"[Title/Abstract]
                OR "buffy coat"[Title/Abstract]
                )
        AND (
                        (
                        "methylation"[Title/Abstract]
                        AND (
                                "epigenetic"[Title/Abstract]
                                OR "epigenome"[Title/Abstract]
                                )
                        )
                OR "CpG"[Title/Abstract]
                OR "450K"[Title/Abstract]
                OR "Infinium"[Title/Abstract]
        )
        AND (
                "marker"[Title/Abstract]
                OR "biomarker"[Title/Abstract]
                OR "diagnosis"[Title/Abstract]
                OR "detection"[Title/Abstract]
                OR "screening"[Title/Abstract]
        )
)
AND (("2011/01/01"[PDat] : "2016/06/31"[PDat]))
```

Table S1 Leukocyte DNA methylation markers in the literature.

Publication	Cancer	Markers
Wu (2016)	Hepatocellular carcinoma	cg12680131, cg22511877
Roos et al. (2016)[a]	Various	cg02444695, cg26079695, cg27094856, cg21046959
Konecny et al. (2016)	Lung cancer	SHOX2
Ho et al. (2015)	Colorectal adenomas	DNMT3B
Guo et al. (2016)	Esophageal squamous cell carcinoma	RASSF2
Joyce et al. (2015)	Various	IFNG, ICAM1, IL6
Cordero et al. (2015)	Colorectal and breast cancer	No hits for CRC
Dauksa et al. (2014)	Gastric cancer	APC, DAPK1, BCL2, CD44, TNFRSF10C, ACIN1
Yang et al. (2015)[a]	Breast cancer	HYAL2
Winham et al. (2014)	Ovarian cancer	cg04389426, cg05674150
Tahara et al. (2013)	Gastric cancer	SFRP1
You et al. (2013)	Nasopharyngeal carcinoma	CDK10
Hajikhan Mirzaei et al. (2012)	Breast cancer	RHOBTB2
Xu et al. (2013)	Breast cancer	cg17378989, cg10237911, cg22385477, cg08287471, cg13905388
Heyn et al. (2013)	Breast cancer	cg15652666
Langevin et al. (2012)	Head and neck cancer	FGD4, SERPINF1, CIAO1, IL27, HYAL2, PLEKHA6
Bosviel et al. (2012)	Breast cancer	BRCA1
You et al. (2012)	Esophageal squamous cell carcinoma	PTPRO
Marsit et al. (2011)	Bladder cancer	NLRP4, BDKRB1, ATG2B, COX7C, ZNF322B, HIGD2A, TBCA, BRD7, PSME2

[a]Performed adjustment for leukocyte composition

References (Supplement)

Bosviel, R., Garcia, S., Lavediaux, G., Michard, E., Dravers, M., Kwiatkowski, F., ... Bernard-Gallon, D.J. (2012). BRCA1 Promoter Methylation in Peripheral Blood DNA Was Identified in Sporadic Breast Cancer and Controls. *Cancer Epidemiology*, *36*(3), e177–182. doi:10.1016/j.canep.2012.02.001

Cordero, F., Ferrero, G., Polidoro, S., Fiorito, G., Campanella, G., Sacerdote, C., ... Naccarati, A. (2015). Differentially Methylated microRNAs in Prediagnostic Samples of Subjects Who Developed Breast Cancer in the European Prospective Investigation into Nutrition and Cancer (EPIC-Italy) Cohort. *Carcinogenesis*, *36*(10), 1144–1153. doi:10.1093/carcin/bgv102

Dauksa, A., Gulbinas, A., Endzinas, Z., Oldenburg, J., & El-Maarri, O. (2014). DNA Methylation at Selected CpG Sites in Peripheral Blood Leukocytes Is Predictive of Gastric Cancer. *Anticancer Research*, *34*(10), 5381–5388.

Guo, W., Dong, Z., Cui, J., Guo, Y., Shen, S., Guo, X., & Kuang, G. (2016). Aberrant Hypermethylation of RASSF2 in Tumors and Peripheral Blood DNA as a Biomarker for Malignant Progression and Poor Prognosis of Esophageal Squamous Cell Carcinoma. *Clinical & Experimental Metastasis*, *33*(1), 73–85. doi:10.1007/s10585-015-9759-5

Hajikhan Mirzaei, Noruzinia, M., Karbassian, H., Shafeghati, Y., Keyhanee, M., & Bidmeshki-Pour, A. (2012). Evaluation of Methylation Status in the 5'UTR Promoter Region of the DBC2 Gene as a Biomarker in Sporadic Breast Cancer. *Cell J*, *14*(1), 19–24.

Heyn, H., Carmona, F.J., Gomez, A., Ferreira, H.J., Bell, J.T., Sayols, S., ... Esteller, M. (2013). DNA Methylation Profiling in Breast Cancer Discordant Identical Twins Identifies DOK7 as Novel Epigenetic Biomarker. *Carcinogenesis*, *34*(1), 102–108. doi:10.1093/carcin/bgs321

Ho, V., Ashbury, J.E., Taylor, S., Vanner, S., & King, W.D. (2015). Gene-Specific DNA Methylation of DNMT3B and MTHFR and Colorectal Adenoma Risk. *Mutation Research/Fundamental and Molecular Mechanisms of Mutagenesis*, *782*, 1–6. doi:10.1016/j.mrfmmm.2015.09.005

Joyce, B.T., Gao, T., Liu, L., Zheng, Y., Liu, S., Zhang, W., ... Hou, L. (2015). Longitudinal Study of DNA Methylation of Inflammatory Genes and Cancer Risk. *Cancer Epidemiology, Biomarkers & Prevention: A Publication of the American Association for Cancer Research, Cosponsored by the American*

Society of Preventive Oncology, *24*(10), 1531–1538. doi:10.1158/1055-9965.EPI-15-0198

Konecny, M., Markus, J., Waczulikova, I., Dolesova, L., Kozlova, R., Repiska, V., ... Majer, I. (2016). The Value of SHOX2 Methylation Test in Peripheral Blood Samples Used for the Differential Diagnosis of Lung Cancer and Other Lung Disorders. *Neoplasma.* Advanced online publication. doi:10.4149/210_150419N208

Langevin, S.M., Koestler, D.C., Christensen, B.C., Butler, R.A., Wiencke, J.K., Nelson, H.H., ... Kelsey, K.T. (2012). Peripheral Blood DNA Methylation Profiles Are Indicative of Head and Neck Squamous Cell Carcinoma: An Epigenome-Wide Association Study. *Epigenetics*, *7*(3), 291–299. doi:10.4161/epi.7.3.19134

Marsit, C.J., Koestler, D.C., Christensen, B.C., Karagas, M.R., Houseman, E.A., & Kelsey, K.T. (2011). DNA Methylation Array Analysis Identifies Profiles of Blood-Derived DNA Methylation Associated With Bladder Cancer. *Journal of Clinical Oncology*, *29*(9), 1133–1139. doi:10.1200/JCO.2010.31.3577

Reinius, L.E., Acevedo, N., Joerink, M., Pershagen, G., Dahlén, S.-E., Greco, D., ... Kere, J. (2012). Differential DNA Methylation in Purified Human Blood Cells: Implications for Cell Lineage and Studies on Disease Susceptibility. *PloS One*, *7*(7), e41361. doi:10.1371/journal.pone.0041361

Roos, L., van Dongen, Jenny, Bell, C.G., Burri, A., Deloukas, P., Boomsma, D.I., ... Bell, J.T. (2016). Integrative DNA Methylome Analysis of Pan-Cancer Biomarkers in Cancer Discordant Monozygotic Twin-Pairs. *Clinical Epigenetics*, *8*(1). doi:10.1186/s13148-016-0172-y

Tahara, T., Maegawa, S., Chung, W., Garriga, J., Jelinek, J., Estécio, M.R.H., ... Issa, J.-P.J. (2013). Examination of Whole Blood DNA Methylation as a Potential Risk Marker for Gastric Cancer. *Cancer Prevention Research (Philadelphia, Pa.)*, *6*(10), 1093–1100. doi:10.1158/1940-6207.CAPR-13-0034

Winham, S.J., Armasu, S.M., Cicek, M.S., Larson, M.C., Cunningham, J.M., Kalli, K.R., ... Goode, E.L. (2014). Genome-Wide Investigation of Regional Blood-Based DNA Methylation Adjusted for Complete Blood Counts Implicates BNC2 in Ovarian Cancer: Ovarian Cancer DNA Methylation Study Implicates BNC2. *Genetic Epidemiology*, *38*(5), 457–466. doi:10.1002/gepi.21815

Wu, H.-C. (2016). Blood DNA Methylation Markers in Prospectively Identified Hepatocellular Carcinoma Cases and Controls from Taiwan. *World Journal of Hepatology*, *8*(5), 301. doi:10.4254/wjh.v8.i5.301

Xu, Z., Bolick, S.C.E., DeRoo, L.A., Weinberg, C.R., Sandler, D.P., & Taylor, J.A. (2013). Epigenome-Wide Association Study of Breast Cancer Using Prospectively Collected Sister Study Samples. *Journal of the National Cancer Institute*, *105*(10), 694–700. doi:10.1093/jnci/djt045

Yang, R., Pfütze, K., Zucknick, M., Sutter, C., Wappenschmidt, B., Marme, F., ... Burwinkel, B. (2015). DNA Methylation Array Analyses Identified Breast Cancer-Associated HYAL2 Methylation in Peripheral Blood. *International Journal of Cancer*, *136*(8), 1845–1855. doi:10.1002/ijc.29205

You, Y., Yang, W., Wang, Z., Zhu, H., Li, H., Lin, C., & Ran, Y. (2013). Promoter Hypermethylation Contributes to the Frequent Suppression of the CDK10 Gene in Human Nasopharyngeal Carcinomas. *Cellular Oncology (Dordrecht)*, *36*(4), 323–331. doi:10.1007/s13402-013-0137-5

You, Y.-J., Chen, Y.-P., Zheng, X.-X., Meltzer, S.J., & Zhang, H. (2012). Aberrant Methylation of the PTPRO Gene in Peripheral Blood as a Potential Biomarker in Esophageal Squamous Cell Carcinoma Patients. *Cancer Letters*, *315*(2), 138–144. doi:10.1016/j.canlet.2011.08.032
